# Dengue Meteorological Determinants during Epidemic and Non-Epidemic Periods in Taiwan

**DOI:** 10.3390/tropicalmed7120408

**Published:** 2022-11-29

**Authors:** Shu-Han You, Szu-Chieh Chen, Yi-Han Huang, Hsin-Chieh Tsai

**Affiliations:** 1 Institute of Food Safety and Risk Management, National Taiwan Ocean University, Keelung City 20224, Taiwan; 2 Department of Public Health, Chung Shan Medical University, Taichung 40201, Taiwan,; 3 Department of Family and Community Medicine, Chung Shan Medical University Hospital, Taichung 40201, Taiwan

**Keywords:** dengue fever, temperature, epidemic, Taiwan, climate, environmental factors

## Abstract

The identification of the key factors influencing dengue occurrence is critical for a successful response to the outbreak. It was interesting to consider possible differences in meteorological factors affecting dengue incidence during epidemic and non-epidemic periods. In this study, the overall correlation between weekly dengue incidence rates and meteorological variables were conducted in southern Taiwan (Tainan and Kaohsiung cities) from 2007 to 2017. The lagged-time Poisson regression analysis based on generalized estimating equation (GEE) was also performed. This study found that the best-fitting Poisson models with the smallest QICu values to characterize the relationships between dengue fever cases and meteorological factors in Tainan (QICu = −8.49 × 10^−3^) and Kaohsiung (−3116.30) for epidemic periods, respectively. During dengue epidemics, the maximum temperature with 2-month lag (β = 0.8400, *p* < 0.001) and minimum temperature with 5-month lag (0.3832, *p* < 0.001). During non-epidemic periods, the minimum temperature with 3-month lag (0.1737, *p* < 0.001) and mean temperature with 2-month lag (2.6743, *p* < 0.001) had a positive effect on dengue incidence in Tainan and Kaohsiung, respectively.

## 1. Introduction

Dengue fever (DF), a virus infection spread by mosquitoes, is most common in urban and suburban areas of tropical and subtropical climates. The incidence of dengue is increasing, and has reached an estimated 390 million annual cases, of which 96 million presents with clinical symptoms [1,2]. It is the most rapidly spreading vector-borne disease and is now endemic in over 100 countries, with half of the world’s population living in a dengue-risk area [3].

Dengue epidemics are known to be related to fluctuations in temperature, precipitation, relative humidity (RH) and socioeconomic factors, such as urbanization and human movement [4,5,6,7,8,9,10,11,12]. Previous study has suggested that a 2 °C increase in temperature would simultaneously lengthen the lifespan of the mosquito and shorten the extrinsic incubation period of dengue virus, resulting in more infected mosquitoes for a longer period of time [13]. Recent research has found that diurnal temperature ranges are more important than average temperature when studying the development and transmission of dengue and malaria parasites [14,15,16]. Precipitation could provide water for breeding because *Aedes* spp. mosquitos prefer artificial containers around human-made environments [10]. However, mosquito survival is also reduced by extreme temperatures and large rainfall events [17].

Taiwan is located in a subtropical and tropical region, with relatively high temperature and RH year-round, which is an ideal condition for mosquitoes that transmit dengue fever [18]. The primary mosquito vector of dengue virus in Taiwan is *Aedes aegypti*, followed by some transmission by *Aedes albopictus* [19]. Dengue fever is a communicable disease that must be reported, and a national web-based communicable diseases surveillance system has been established since 1997 [20]. According to the reports from Taiwan Centers for Disease Control, the main epidemic areas of dengue fever from 1998 to 2021 are located in southern Taiwan, including Kaohsiung City (45,658 indigenous dengue cases), Tainan City (26,580 indigenous dengue cases), and Pingtung County (2058 indigenous dengue cases) [20]. Over the last decade, two consecutive severe dengue epidemics occurred in 2014 and 2015 in southern Taiwan [21,22,23,24,25,26]. The death rate in 2015 dengue hemorrhagic fever (DHF)/dengue shock syndrome (DSS) cases was higher than in previous dengue epidemics or outbreaks [21]. Evidence-based study indicated that the majority of population in Taiwan born after 1940 is naïve to dengue virus and the prevalence of IgG antibody against dengue virus rises with age [27].

Associations between temperature, RH, precipitation and dengue transmission have been mentioned in previous studies but there is no consistent conclusion have been found [18,28,29]. Several studies have been conducted to explore the association between climate variables and dengue fever with lagged effect during epidemic [6,30,31]. The time lagged effect could be caused by the lifespan of the mosquito and the incubation period of dengue virus [31].

Therefore, this study aims to link meteorological factors with weekly incidence rate of dengue fever to investigate the key determinants during epidemic and non-epidemic periods. We also try to explore the difference of time-lag effects of meteorological factors during dengue-epidemic (2014–2015) and non-epidemic periods (2007–2013 and 2016–2017) by statistical models.

## 2. Materials and Methods

### 2.1. Surveillance Data

The Taiwan National Infectious Disease Statistics System (Taiwan NIDSS) was developed by Taiwan Center of Disease Control (Taiwan CDC) for dengue fever [32]. The Taiwan NIDSS provides statistical information regarding dengue fever surveillance data, including trend, map, figures, period comparison, and imported cases. Study areas focus on the highest epidemic region in Kaohsiung and Tainan cities of southern Taiwan.

Because of the major dengue epidemic that occurred in southern Taiwan, and the most recent clustered epidemic area that appeared in Tainan. During epidemic periods in 2014 and 2015, there were 15,496 and 43,419 indigenous confirmed dengue cases, respectively. All weekly number of confirmed dengue cases were provided by Taiwan CDC [32] and included eleven consecutive years from January 2007 to December 2017. Weekly-based incidence rates per 100,000 population were estimated from the year-week indigenous confirmed dengue cases over the specific year-end population size.

### 2.2. Meteorological Data

All weekly meteorological data for Tainan and Kaohsiung from 2007 to 2017 were derived from observations at four (Xinying, Shanhua, Annan, and Tainan stations) and eleven monitor stations (Qiaotou, Nanzi, Renwu, Zuoying, Qianjin, Qianzhen, Fuxing, Fongshan, Xiangang, Linyuan, and Dalia stations) of Taiwan Environmental Protection Agency [33] and Central Weather Bureau [34]. The study data included the weekly maximum (max.), mean, and minimum (min.) temperatures (°C), relative humidity (%), and rainfall intensity (mm). The weekly max., mean, and min. temperatures were defined as the weekly average of the daily max., mean, and min. temperatures, respectively.

### 2.3. Statistics Analysis

We began by investigating the overall relationships between dengue incidence rates and meteorological data (min., mean, and max. temperatures, RH and amount of rainfall). According to surveillance system, the years 2014–2015 were defined as dengue epidemic periods, while the years 2007–2013 and 2016–2017 were designated as non-epidemic periods. We used cross-correlation to estimate the associations between the time-lags of the meteorological variables and the incidence of dengue fevers in Tainan and Kaohsiung for dengue epidemic and non-epidemic periods, respectively. The lagged-time Poisson regression analysis was performed [35]. Meteorological variables with *n*-month lag time were independent variables while dengue incidence was a dependent variable. The subscript of *t-n* refers to each meteorological variable with *n*-month lag time in a basic multivariate Poisson regression model. The following is a basic multivariate Poisson regression model,

(1) $$\ln\left(Y_{t}\right)=\beta_{0}+\beta_{1}T_{\max,t-n}+\beta_{2}T_{\min,t-n}+\beta_{3}T_{\mathrm{mean},t-n}+\beta_{4}{\mathrm{Rain}}_{t-n}+\beta_{5}{\mathrm{RH}}_{t-n}$$

where *Y_t_* is the incidence of confirmed dengue cases at time *t*, *β*_0_ is the intercept, *β*_1_ through *β*_5_ represent coefficients, *T*_max_, *T*_min_ and *T*_mean_ are the weekly max., min., and mean temperatures, respectively (°C), Rain represents the rainfall intensity (mm), RH represents the relative humidity (%), and *t*−*n* in the subscript represents the *n*-month lag time. Based on the quasi-likelihood-based information criterion (QICu) statistic, the most parsimonious model was selected [35,36]. The best fitting model with the smallest QICu was preferred. The lagged-time Poisson regression analyses based on generalized estimating equation (GEE) were performed by using SAS Version 9.1.3 for Windows (SAS Institute Inc., Cary, NC, USA).

## 3. Results

### 3.1. Data Description

**Figure 1:**
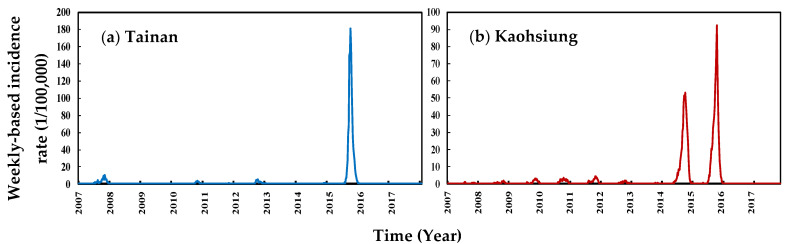
Weekly-based incidence rate in (**a**) Tainan and (**b**) Kaohsiung from 2007–2017 years.

In Tainan, the mean and max. weekly incidence rates were 2.41 and 181.54 per 100,000 people, respectively, whereas in Kaohsiung, they were nearly 2.45 and 92.52 per 100,000 people in Kaohsiung. The dengue fever epidemics in these two areas reached their peak in 2015. There are 19,769 and 15,013 reported dengue cases among the population size of 2,778,276 and 2,777,873 in 2015 in Kaohsiung and Tainan, respectively (Figure 1).

**Figure 2:**
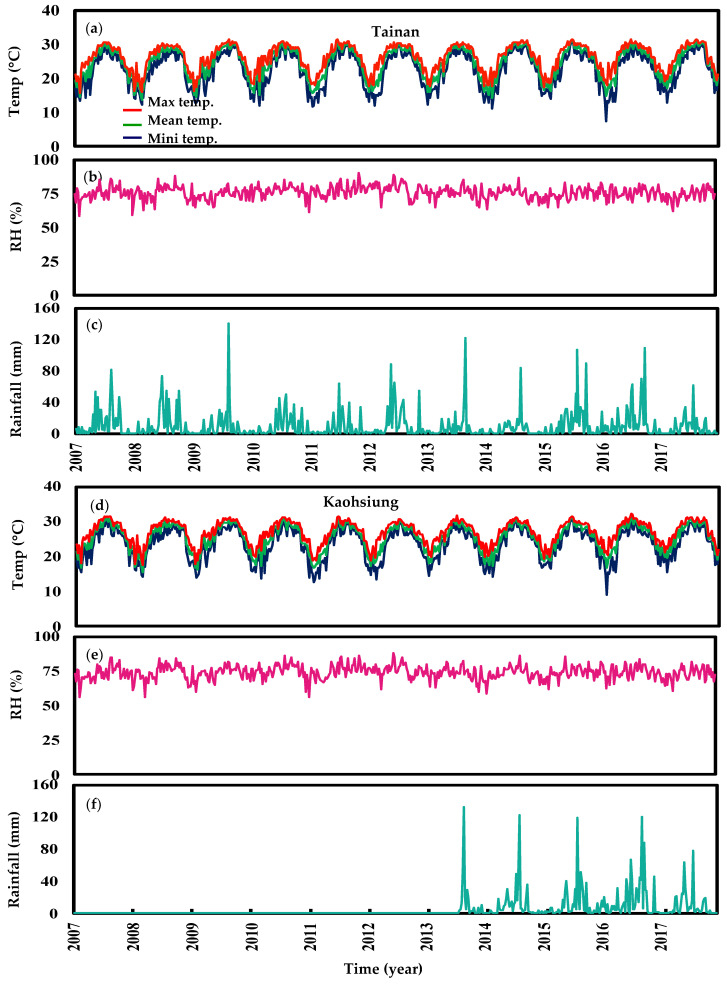
(**a**,**d**) Weekly maximum, mean, and minimum temperatures (°C), (**b**,**e**) relative humidity (RH) (%) and (**c**,**f**) rainfall (mm) in Tainan and Kaohsiung from 2007–2017 years, respectively.

Figure 2 illustrates meteorological data for the two areas from 2007 to 2017. Tainan has a tropical climate, with a mean annual temperature of 24.62 °C, the maximum temperature of 32.95 °C in August, the lowest temperature of 7.43 °C in January, a mean yearly RH of nearly 75.75%, and a mean weekly rainfall of 10.64 mm. However, the results in Kaohsiung reveal a tropical climate with a mean yearly temperature of 25.47 °C, a maximum temperature of 33.01 °C in summer, a minimum temperature of 7.25 °C in winter, a mean yearly RH of 74.09%, and a mean weekly rainfall of 11.41 mm. Statistical description for each during 2007 to 2017 were also presented. We listed the mean, standard deviation (SD), min., Q1, Q2, Q3, and max. for each environmental monitor station (EMS), as well as meteorological data such as min. temp., mean temp., max. temp., RH, and rainfall (Tables S1–S3).

### 3.2. Trend Analysis

**Figure 3:**
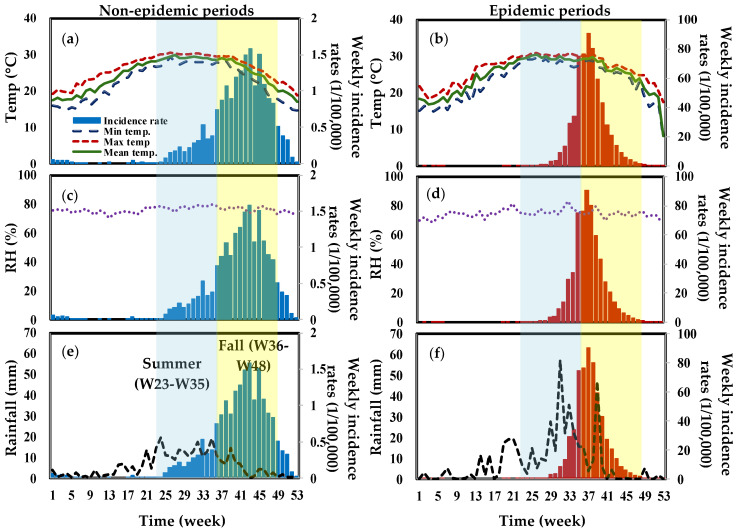
The overall trend analysis of dengue weekly incidence rates and meteorological variables of weekly (**a**,**b**) mean, maximum and minimum temperatures (°C), (**c**,**d**) RH (%) as well as (**e**,**f**) rainfall (mm) during non-epidemic and epidemic periods, respectively, in Tainan.

**Figure 4:**
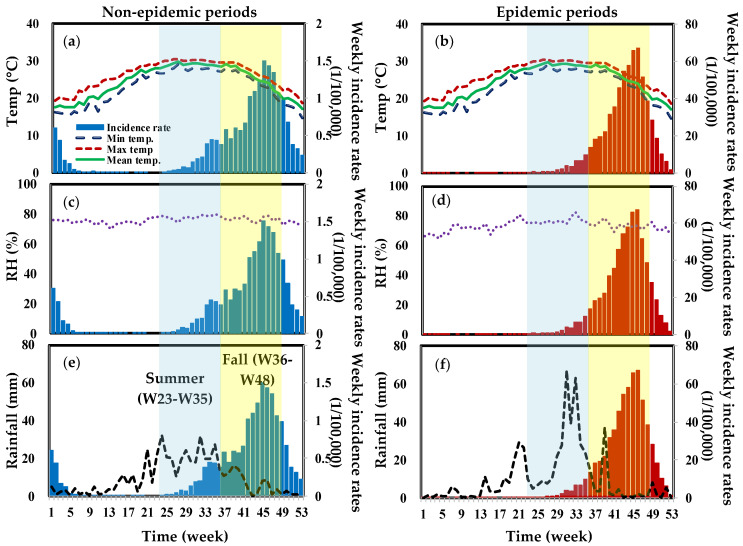
The overall trend analysis of dengue weekly incidence rates and meteorological variables of weekly (**a**,**b**) mean, maximum and minimum temperatures (°C), (**c**,**d**) RH (%) as well as (**e**,**f**) rainfall (mm) during non-epidemic and epidemic periods, respectively, in Kaohsiung.

We distribute meteorological data in Tainan and Kaohsiung into epidemic and non-epidemic periods. Figure 3 and Figure 4 show a trend analysis of weekly mean, maximum, and minimum temperatures, RH, and rainfall in Tainan and Kaohsiung from 2007 to 2017. The mapping of average weekly rainfall and average weekly incidence rates revealed significant time lag effects (Figure 3f and Figure 4f). Rainfall patterns are similar in both areas. Our data show that there was heavy rainfall prior to the peak of the dengue epidemic, with the highest recorded in Kaohsiung reaching 60 mm.

Spearman’s rank correlation coefficient fitted to data indicate the monthly time-lag effects in the mean, maximum, and minimum temperatures, rainfall, and RH in two study areas during non-epidemic and epidemic periods (Table 1). The results show that a 3–4 months lag in temperatures explained 54–56% (non-epidemic period) and 22–29% (epidemic period) of the variability in dengue incidences in two time periods in Tainan region. However, in Kaohsiung region, higher significant trends in the temperature (*r* = 0.54–0.64) and rainfall (*r* = 0.53–0.55) were found 5 months lag, yet there are lower positive trends revealed in the RH. Rainfall with a 1-month lag (Tainan) and 3-month lag (Kaohsiung) also had an impact on dengue incidence variability (*r* = 0.46 and 0.55) (Table 1).

### 3.3. Lagged-Time Poisson Regression Analysis

The time lag effects were then incorporated into the Poisson GEE model to determine the dengue incidence attributable to meteorological factors. Table 2 presents the best-fitting models with the smallest QICu values for characterizing the relationships between weekly dengue fever cases and meteorological factors in Tainan (QICu = −8.49 × 10^−3^) and Kaohsiung (QICu = −3116.30) for epidemic periods (2014–2015), respectively. The most positive effects on dengue incidence were the maximum temperature at a lag of 2-month in Tainan (β = 0.8400, *p* < 0.001) and minimum temperature at a lag of 5-month in Kaohsiung (0.3832, *p* < 0.001) for epidemic periods, respectively (Table 2).

On the other hand, the best-fitting models with the smallest QICu values were 63.17 and 69.24 in Tainan and Kaohsiung for non-epidemic periods (2007–2013 and 2016–2017), respectively (Table 2). The most positive effects on dengue incidence were minimum temperature at a lag of 3-month in Tainan (0.1737, *p* < 0.001) and mean temperature at a lag of 2-month in Kaohsiung (2.6743, *p* < 0.001) for non-epidemic periods, respectively (Table 2).

## 4. Discussion

This study linked meteorological factors and epidemiological data to characterize the patterns in southern Taiwan from 2007 to 2017. We try to explore the difference and time-lag effect by meteorological factors during epidemic (2014–2015) and non-epidemic periods (2007–2013 and 2016–2017) by statistical models. Several aspects of dengue fever risk in southern Taiwan are revealed by our analysis. The major findings are as follows: (i) the effect of temperature variation may have a significant impact on dengue incidence rates than the other factors when compared with non-epidemic (2007–2013 and 2016–2017) and epidemic periods (2014–2015), (ii) the results show that 3–4 months lag in temperatures explained 54–56% (non-epidemic periods) and 22–29% (epidemic periods) of the variability in dengue incidences in two time periods in Tainan region, and (iii) For epidemic periods, the maximum temperature with 2-month lag (β = 0.8400, *p* < 0.001) and minimum temperature with 5-month lag (0.3832, *p* < 0.001); for non-epidemic periods minimum temperature with 3-month lag (0.1737, *p* < 0.001) and mean temperature with 2-month lag (2.6743, *p* < 0.001) had a positive effect on dengue incidence in Tainan and Kaohsiung, respectively.

According to the meta-analysis study [4], both temperature and precipitation increase the risk of dengue fever in tropical and subtropical regions. Temperature (RR = 1.13, 95% CI: 1.12, 1.15) and precipitation (RR = 1.01, 95% CI: 1.01, 1.01) were also found to have a statistically significant correlation with the risk of dengue fever [4]. Lee et al. [8] found that temperature and precipitation were positively associated with the occurrence of disease from 1994 to 2013 in Vietnam which was consistent with findings from the previous studies [37,38,39]. Huang et al. [40] found that the relative risk of dengue fever increased when the weekly average temperature was more than 15 °C at lagged weeks 5 to 18 in a study of meteorological factors affecting dengue fever in Kaohsiung in 2015. Yuan et al. [41] found that dengue transmission has a positive relationship with the minimum temperature predictors during the early summer while a negative relationship with all the maximum 24-h rainfall predictors during the early epidemic phase of dengue outbreaks. In our study, minimum and mean temperatures with time lag shown the significantly effects on dengue incidence in Tainan and Kaohsiung at non-epidemic periods (Table 2); However, in epidemic periods, maximum and minimum temperature shown the significantly factors (Table 2).

In this study, temperature with time lag shown the different effects on dengue incidence in Tainan during the epidemic and non-epidemic periods; however, rainfall with time lag shown the significantly positive effect on dengue incidence in Kaohsiung city during the epidemic and non-epidemic periods (Table 2). Previous studies have also described the high temperatures and heavy rains in 2014 and 2015 in southern Taiwan [26,42]. Furthermore, one study mentioned the dengue outbreak in Kaohsiung city in 2014 might be due to the natural gas explosion incident on 1 August 2014 [42]. After the explosion, this situation created an ideal environment for *Aedes* mosquitoes [43,44]. The outbreak in 2015, Tainan city was the original division of the outbreak, and it had the highest prevalence rate of dengue. The reasons for this outbreak may include geographical location, high population density, and inadequate control of dengue outbreaks [26].

The typhoon season from July to September has always been the main source of rainfall in Taiwan. During the most intense typhoons, a total of more than 1000 mm of rain has fallen in a few days. According to the Central Weather Bureau [34], different typhoon tracks affect the magnitude of rainfall in different regions of Taiwan. During the epidemic periods, Taiwan experienced two moderate typhoons and rain incidents occurred along with the southeast airflow. In 2014, southern Taiwan continued to fall from 7 August to 12 August. It is shown that the 24-h cumulative rainfall in Tainan’s Administrative Westport District and Anding District exceeded 350 mm, reaching super heavy rain. The accumulated rainfall in 24 h exceeded 200 mm, reaching the standard of heavy rain [45]. During typhoon season, the heavy rainfall causes a hot, wet environment with a high RH that is ideal for mosquito breeding [44]. Even though our research showed that the RH with time lag had different effects on dengue incidence in Tainan and Kaohsiung during the epidemic and non-epidemic periods.

The main dengue control strategies in Taiwan are to eliminate vector breeding sources and effectively reduce vector (mosquito) density. Three-stage prevention strategy for dengue epidemic control have been implicated. Initial control measures include source reduction and control of vector populations. Secondary measures include disease surveillance and emergency/precautionary mechanisms, and tertiary prevention includes mortality control [32].

A few limitations of this study warrant mention. First, this study only considers the major metrological factors, such as the weekly max., mean, and min. temperatures, rainfall intensity, and RH. The following variables were not considered: daily maximum rainfall, ELSO, population density, mosquito density, or human activity. With regards to the human populations, this study assumes a homogenous population even though exposure to mosquitoes and dengue risk are known to vary because of socioeconomic factors [46], race or ethnicity, and lifestyle choices such as time spent outdoors [47]. Moreover, the long-term investigation of the Breteau Index or Contain Index in Taiwan was also limited. The temporal and spatial distributions of the mosquito population or host population were not presented.

## 5. Conclusions

This study has identified the weekly maximum (max.), mean, and minimum (min.) temperatures (°C), relative humidity (%), and rainfall intensity (mm) as potential contributors, with max. temperature at lag 2 months and lag 5 months being the most significant, for dengue incidence in Tainan and Kaohsiung, respectively. Therefore, the results of this study suggest that vector control interventions in dengue epidemics are more effective at the early stage of the epidemic.

## Figures and Tables

**Table 1:** Results of Spearman’s coefficient of rank correlation for time-lag effects.

Non-Epidemic Periods						Epidemic Periods					
Time-Lag (Months)	Mean Temp. (°C)	Max. Temp. (°C)	Min. Temp. (°C)	Rainfall (mm)	RH (%)	Time-Lag (Months)	Mean Temp. (°C)	Max. Temp. (°C)	Min. Temp. (°C)	Rainfall (mm)	RH (%)
	Tainan										
0	0.07	0.05	0.05	4 × 10^−5^	0.08		0.16	0.16	0.17	0.08	0.09
1	0.30	0.26	0.28	0.15	0.23	1	0.21	0.22	0.20	0.46	0.19
2	0.49	0.44	0.49	0.48	0.41	2	0.24	0.22	0.25	0.21	0.07
3	0.55	0.52	0.56	0.56	0.34	3	0.25	0.22	0.29	0.005	0.02
4	0.56	0.54	0.56	0.41	0.17	4	0.16	0.15	0.16	0.02	0.17
5	0.44	0.45	0.44	0.20		5	0.05	0.07	0.03		
6	0.21	0.24	0.21			6	0.01	0.0002	0.04		
	Kaohsiung										
0	0.0027	0.01	1 × 10^−5^	0.04	0.04		0.0016	0.0002	0.004	0.04	0.0002
1	0.14	0.11	0.17	0.01	0.12	1	0.06	0.04	0.07	0.001	0.02
2	0.39	0.34	0.41	0.20	0.30	2	0.20	0.19	0.20	0.14	0.16
3	0.53	0.48	0.54	0.51	**0.47**	3	0.31	0.30	0.31	**0.55**	**0.35**
4	0.63	0.59	0.64	**0.53**	0.28	4	0.48	0.44	0.49	0.28	0.30
5	**0.64**	**0.62**	**0.64**	0.40		5	**0.58**	**0.54**	**0.61**		
6	0.51	0.53	0.49			6	0.42	0.42	0.40		

The largest value of the correlation coefficient obtained from the cross-correlation analyses is indicated by the bold face. Abbreviations: Min. Temp. is the minimum temperature; mean Temp. is the mean temperature; Max. Temp. is the maximum temperature; RH is the relative humidity.

**Table 2:** The statistics of the best Poisson models based on generalized estimating equation (GEE) of the weekly dengue incidence rate (2007–2017) on meteorological factors in Tainan and Kaohsiung during epidemic and non-epidemic periods.

Tainan	Non-Epidemic Periods		Tainan	Epidemic Periods	
	*β*	*p*		*β*	*p*
Mini temp. (Lag 3) ^b^	0.1737	***	Mini temp. (Lag 4)	0.2474	**
Mean temp. (Lag 5)	0.1041	**	Mini temp. (Lag 5)	−0.2164	***
Max. temp.	0.1396	***	Max. temp. (Lag 2)	0.8400	***
RH	0.1099	*	Max. temp. (Lag 3)	0.8289	***
RH (Lag 2)	−0.0699	***	RH	−0.0822	***
Rainfall	−0.0928	***	RH (Lag 1)	0.1360	***
Rainfall (Lag 2)	0.0542	***	RH (Lag 4)	−0.1221	***
			Rainfall (Lag 1)	0.0335	***
			Rainfall (Lag 3)	−0.0404	***
QICu	63.17		QICu	−8.49 × 10^−3^	
**Kaohsiung**	**Non-Epidemic Periods**		**Kaohsiung**	**Epidemic Periods**	
	***β***	***p***		***β***	***p***
Mini temp. (Lag 1)	0.6896	***	Mini temp. (Lag 5)	0.3832	***
Mini temp. (Lag 2)	−0.8118	***	RH (Lag 1)	−0.0723	***
Mean temp. (Lag 1)	−0.7499	***	Rainfall (Lag 1)	0.0127	***
Mean temp. (Lag 2)	2.6743	***	Rainfall (Lag 2)	0.0139	***
Mean temp. (Lag 4)	0.1532	*			
Mean temp. (Lag 5)	−0.1665	**			
Mean temp. (Lag 6)	0.1678	***			
Max. temp. (Lag 2)	−1.8695	***			
Rainfall (Lag 3)	0.0122	*			
QICu	69.24		QICu	−3116.30	

‘***’ 0.001 ‘**’ 0.01 ‘*’ 0.05. ^b^ Lag 1—5” indicates the time-lag in month. Abbreviations: Min. Temp. is the minimum temperature; mean Temp. is the mean temperature; Max. Temp. is the maximum temperature; RH is the relative humidity.

## Data Availability

All data generated or analyzed during this study are included in this published article and in the Supplementary Materials.

## Supplementary Materials

The following supporting information can be downloaded at: https://www.mdpi.com/article/10.3390/tropicalmed7120408/s1, Table S1: Statistical description of each meteorological data during 2007 to 2017 in Tainan’s Environmental Monitor Station (EMS); Table S2: Statistical description of each meteorological data during 2007 to 2017 in Kaohsiung’s Environmental Monitor Station (EMS); Table S3: Comparison of the mean estimations and standard deviation of meteorological factors in epidemic and non-epidemic periods.

## References

[1] Bhatt S., Gething P.W., Brady O.J., Messina J.P., Farlow A.W., Moyes C.L., Drake J.M., Brownstein J.S., Hoen A.G., Sankoh O. (2013). The global distribution and burden of dengue. Nature.

[2] Stanaway J.D., Shepard D.S., Undurraga E.A., Halasa Y.A., Coffeng L.E., Brady O.J., Hay S.I., Bedi N., Bensenor I.M., Castañeda-Orjuela C.A. (2016). The global burden of dengue: An analysis from the Global Burden of Disease Study 2013. Lancet Infect. Dis..

[3] World Health Organization (WHO) (2022). Dengue and Severe Dengue.

[4] Li Y., Dou Q., Lu Y., Xiang H., Yu X., Liu S. (2020). Effects of ambient temperature and precipitation on the risk of dengue fever: A systematic review and updated meta-analysis. Environ. Res..

[5] Akter R., Hu W., Gatton M., Bambrick H., Naish S., Tong S. (2020). Different responses of dengue to weather variability across climate zones in Queensland, Australia. Environ. Res..

[6] Xiao J., Liu T., Lin H., Zhu G., Zeng W., Li X., Zhang B., Song T., Deng A., Zhang M. (2018). Weather variables and the El Niño Southern Oscillation may drive the epidemics of dengue in Guangdong Province, China. Sci. Total Environ..

[7] Chuang T.W., Chaves L.F., Chen P.J. (2017). Effects of local and regional climatic fluctuations on dengue outbreaks in southern Taiwan. PLoS ONE.

[8] Lee H.S., Nguyen-Viet H., Nam V.S., Lee M., Won S., Duc P.P., Grace D. (2017). Seasonal patterns of dengue fever and associated climate factors in 4 provinces in Vietnam from 1994 to 2013. BMC Infect. Dis..

[9] Guzman M.G., Harris E. (2015). Dengue. Lancet.

[10] Morin C.W., Comrie A.C., Ernst K. (2013). Climate and dengue transmission: Evidence and implications. Environ. Health Perspect..

[11] Liu X., Liu K., Yue Y., Wu H., Yang S., Guo Y., Ren D., Zhao N., Yang J., Liu Q. (2021). Determination of factors affecting dengue occurrence in representative areas of China: A principal component regression analysis. Front. Public Health.

[12] Tuladhar R., Singh A., Varma A., Choudhary D.K. (2019). Climatic factors influencing dengue incidence in an epidemic area of Nepal. BMC Res. Notes.

[13] Focks D.A., Barrera R. (2007). Dengue Transmission Dynamics: Assessment and Implications for Control.

[14] Zhao X., Chen F., Feng Z., Li X., Zhou X.H. (2014). Characterizing the effect of temperature fluctuation on the incidence of malaria: An epidemiological study in south-west China using the varying coefficient distributed lag non-linear model. Malar. J..

[15] Lambrechts L., Paaijmans K.P., Fansiri T., Carrington L.B., Kramer L.D., Thomas M.B., Scott T.W. (2011). Impact of daily temperature fluctuations on dengue virus transmission by *Aedes aegypti*. Proc. Natl. Acad. Sci. USA.

[16] Paaijmans K.P., Read A.F., Thomas M.B. (2009). Understanding the link between malaria risk and climate. Proc. Natl. Acad. Sci. USA.

[17] Colón-González F.J., Fezzi C., Lake I.R., Hunter P.R. (2013). The effects of weather and climate change on dengue. PLoS Negl. Trop. Dis..

[18] Wu P.C., Guo H.R., Lung S.C., Lin C.Y., Su H.J. (2007). Weather as an effective predictor for occurrence of dengue fever in Taiwan. Acta Trop..

[19] Yang C.F., Hou J.N., Chen T.H., Chen W.J. (2014). Discriminable roles of *Aedes aegypti* and *Aedes albopictus* in establishment of dengue outbreaks in Taiwan. Acta Trop..

[20] Taiwan Center for Disease Control (2015). Taiwan National Infectious Disease Statistics System for Dengue Virus.

[21] Lee J.C., Cia C.T., Lee N.Y., Ko N.Y., Chen P.L., Ko W.C. (2022). Causes of death among dengue patients causes of death among hospitalized adults with dengue fever in Tainan, 2015: Emphasis on cardiac events and bacterial infections. J. Microbiol. Immunol. Infect..

[22] Wang S.F., Chang K., Loh E.W., Wang W.H., Tseng S.P., Lu P.L., Chen Y.H., Chen Y.M.A. (2016). Consecutive large dengue outbreaks in Taiwan in 2014–2015. Emerg. Microbes Infect..

[23] Wang S.F., Wang W.H., Chang K., Chen Y.H., Tseng S.P., Yen C.H., Wu D.C., Chen Y.M.A. (2016). Severe Dengue Fever Outbreak in Taiwan. Am. J. Trop. Med. Hyg..

[24] Wang W.-H., Lin C.-Y., Chang K., Urbina A.N., Assavalapsakul W., Thitithanyanont A., Lu P.-L., Chen Y.-H., Wang S.-F. (2019). A clinical and epidemiological survey of the largest dengue outbreak in Southern Taiwan in 2015. Int. J. Infect. Dis..

[25] Tsai J.-J., Liu C.-K., Tsai W.-Y., Liu L.-T., Tyson J., Tsai C.-Y., Lin P.-C., Wang W.-K. (2018). Seroprevalence of dengue virus in two districts of Kaohsiung City after the largest dengue outbreak in Taiwan since World War II. PLoS Negl. Trop. Dis..

[26] Hsu J.C., Hsieh C.L., Lu C.Y. (2017). Trend and geographic analysis of the prevalence of dengue in Taiwan, 2010–2015. Int. J. Infect. Dis..

[27] Lee Y.H., Hsieh Y.C., Chen C.J., Lin T.Y., Huang Y.C. (2021). Retrospective Seroepidemiology study of dengue virus infection in Taiwan. BMC Infect. Dis..

[28] Chen S.C., Liao C.M., Chio C.P., Chou H.H., You S.H., Cheng Y.H. (2010). Lagged temperature effect with mosquito transmission potential explains dengue variability in southern Taiwan: Insights from a statistical analysis. Sci. Total Environ..

[29] Chien L.C., Yu H.L. (2014). Impact of meteorological factors on the spatiotemporal patterns of dengue fever incidence. Environ. Int..

[30] Jain R., Sontisirikit S., Iamsirithaworn S., Prendinger H. (2019). Pridection of dengue outbreaks based on disease surveillance, meteorological nad socio-economic data. BMC Infect. Dis..

[31] Yuan H.Y., Liang J., Lin P.S., Sucipto K., Tsegaye M.M., Wen T.H., Pfeiffer S., Pfeiffer D. (2020). The effects of seasonal climate variability on dengue annual incidence in Hong Kong: A modelling study. Sci. Rep..

[32] Taiwan Center of Disease Control. https://nidss.cdc.gov.tw/Home/Index?op=4.

[33] Taiwan Environmental Protection Agency. https://www.epa.gov.tw/.

[34] Central Weather Bureau Typhoon Database. https://rdc28.cwb.gov.tw/TDB/public/warning_typhoon_list/.

[35] Lu L., Lin H., Tian L., Yang W., Sun J., Liu Q. (2009). Time series analysis of dengue fever and weather in Guangzhou, China. BMC Public Health.

[36] Pan W. (2001). Akaike’s information criterion in generalized estimating equations. Biometrics.

[37] Promprou S., Jaroensutasinee M., Jaroensutasinee K. (2005). Climatic factors affecting dengue haemorrhagic fever incidence in southern Thailand. Dengue Bull..

[38] Wiwanitkit V. (2006). An observation on correlation between rainfall and the prevalence of clinical cases of dengue in Thailand. J. Vector Borne Dis..

[39] Pham H.V., Doan H.T., Phan T.T., Minh N.N. (2011). Ecological factors associated with dengue fever in a Central Highlands province, Vietnam. BMC Infect. Dis..

[40] Huang C.H., Lin C.Y., Yang C.Y., Chan T.C., Chiang P.H., Chen Y.H. (2021). Relationship between the incidence of dengue virus transmission in traditional market and climatic conditions in Kaohsiung city. Can. J. Infect. Dis. Med. Microbiol..

[41] Yuan H.Y., Wen T.H., Kung Y.H., Tsou H.H., Chen C.H., Chen L.W., Lin P.S. (2019). Prediction of annual dengue incidence by hydro-climatic extremes for southern Taiwan. Int. J. Biometeorol..

[42] Wang S.F., Chang K., Lu R.W., Wang W.H., Chen Y.H., Chen M., Wu D.C., Chen Y.M.A. (2015). Large Dengue virus type 1 outbreak in Taiwan. Emerg. Microbes Infect..

[43] Lwin M.O., Vijaykumar S., Fernando O.N.N., Cheong S.A., Rathnayake V.S., Lim G., Theng Y.-L., Chaudhuri S., Foo S. (2014). A 21st century approach to tackling dengue: Crowdsourced surveillance, predictive mapping and tailored communication. Acta Trop..

[44] Lai L.W. (2011). Influence of environmental conditions on asynchronous outbreaks of dengue disease and increasing vector population in Kaohsiung, Taiwan. Int. J. Environ. Health Res..

[45] (2021). National Science and Technology Center for Disaster Reduction. https://den.ncdr.nat.gov.tw/1132/1188/.

[46] Reiter P., Lathrop S., Bunning M., Biggerstaff B., Singer D., Tiwari T., Baber L., Amador M., Thirion J., Hayes J. (2003). Texas lifestyle limits transmission of dengue virus. Emerg. Infect. Dis..

[47] Gubler D.J. (1998). Dengue and dengue hemorrhagic fever. Clin. Microbiol. Rev..

