# Supplementary material for: Dengue Meteorological Determinants during Epidemic and Non-Epidemic Periods in Taiwan
Source: Trop Med Infect Dis. 2022 Nov 29;7(12):408. doi: 10.3390/tropicalmed7120408 (PMC9785930; doi:10.3390/tropicalmed7120408)
Supplement: Supplementary file 1 [file tropicalmed-07-00408-s001.zip › tropicalmed-2039669-supplementary.pdf]

Tropical Medicine and Infectious Disease

MDPI

Supplementary Materials:Dengue Meteorological Determinants during Epidemic and Non-Epidemic Periods in Taiwan

Table S1. Statistical description for each meteorological data during 2007 to 2017 in Tainan's Environmental Monitor Station (EMS).

Variables		Tainan				Tainan Average
		Tainan	Annan	Shanhua	Xinying	
Min. temp. (°C)	AVG	22.77	22.85	22.66	22.52	22.70
	SD	5.32	5.36	5.02	5.28	5.24
	Min	6.48	7.40	9.22	6.60	7.43
	Q1	18.04	17.97	18.26	17.85	18.03
	Q2	24.26	24.48	24.28	23.68	24.17
	Q3	27.24	27.38	26.85	27.06	27.13
	Max	31.10	31.76	30.08	30.69	30.91
Mean temp. (°C)	AVG	24.70	24.84	24.49	24.45	24.62
	SD	4.51	4.66	4.33	4.55	4.51
	Min	13.11	14.07	13.46	13.43	13.52
	Q1	20.75	20.67	20.78	20.40	20.65
	Q2	25.82	26.02	25.77	25.44	25.76
	Q3	28.59	28.94	28.26	28.43	28.56
	Max	31.42	31.96	30.86	31.18	31.35
Max. temp. (°C)	AVG	26.39	26.58	26.11	26.18	26.31
	SD	3.89	4.10	3.79	3.98	3.94
	Min	15.35	15.51	15.60	15.51	15.49
	Q1	23.44	23.46	23.40	23.19	23.37
	Q2	27.24	27.52	27.26	27.05	27.27
	Q3	29.63	30.15	29.35	29.57	29.68
	Max	35.16	32.78	32.00	31.84	32.95
RH (%)	AVG	74.60	74.40	78.19	75.83	75.75
	SD	5.97	4.97	5.37	5.34	5.41
	Min	53.71	55.51	59.36	57.07	56.41
	Q1	70.88	71.37	75.00	72.45	72.43
	Q2	74.83	74.50	78.55	75.96	75.96
	Q3	78.55	77.75	81.83	79.21	79.33
	Max	91.99	87.92	93.28	92.45	91.41
Rainfall (mm)	AVG	9.45	8.78	11.68	12.65	10.64
	SD	18.68	16.75	24.61	21.98	20.51
	Min	0.00	0.00	0.00	0.00	0.00
	Q1	0.00	0.00	0.00	0.00	0.00
	Q2	1.50	1.50	2.50	4.00	2.38
	Q3	11.00	11.21	11.50	14.63	12.08
	Max	131.63	121.10	204.50	152.50	152.43

Abbreviations: AVG is the average; SD is the standard deviation; Min is the minimum; Q1 is the quartile 1; Q2 is the quartile 2; Q3 is the quartile 3; Max is the maximum; Min. Temp. is the minimum temperature; mean Temp. is the mean temperature; Max. Temp. is the maximum temperature; and RH is the relative humidity.

Table S2. Statistical description for each meteorological data during 2007 to 2017 in Kaohsiung's Environmental Monitor Station (EMS).

Variables		Kaohsiung Average
Min. temp. (°C)	AVG	23.68
	SD	4.73

	Min	7.25
	Q1	19.67
	Q2	25.15
	Q3	27.50
	Max	31.28
Mean temp. (°C)	AVG	25.47
	SD	3.99
	Min	15.34
	Q1	22.14
	Q2	26.63
	Q3	28.82
	Max	31.76
Max. temp. (°C)	AVG	27.03
	SD	3.45
	Min	17.33
	Q1	24.55
	Q2	27.84
	Q3	29.86
	Max	33.01
RH (%)	AVG	74.09
	SD	5.67
	Min	53.21
	Q1	70.58
	Q2	74.31
	Q3	77.86
	Max	91.07
Rainfall (mm)	AVG	11.41
	SD	22.67
	Min	0.06
	Q1	0.30
	Q2	2.34
	Q3	11.81
	Max	160.98

Abbreviations: AVG is the average; SD is the standard deviation; Min is the minimum; Q1 is the quartile 1; Q2 is the quartile 2; Q3 is the quartile 3; Max is the maximum; Min. Temp. is the minimum temperature; mean Temp. is the mean temperature; Max. Temp. is the maximum temperature; and RH is the relative humidity.

Table S3. Comparison the mean estimations and standard deviation of meteorological factors during epidemic and non-epidemic periods.

**Variables**	**Tainan**		**Kaohsiung**	
	**Non-Epidemic periods**	**Epidemic periods**	**Non-Epidemic periods**	**Epidemic periods**
Min. Temp. (°C)	22.61±5.24	23.12±5.28	23.61±4.74	23.97±4.68
Mean Temp. (°C)	24.58±4.47	24.83±4.72	25.46±3.97	25.51±4.09
Max. Temp. (°C)	26.29±3.90	26.43±4.15	27.04±3.44	26.98±3.49
RH (%)	76.03±5.46	74.36±4.50	74.23±5.74	73.45±5.14
Rainfall (mm)	9.90±17.98	11.57±21.85	11.62±21.82	9.58±20.75

Abbreviations: Min. Temp. is the minimum temperature; mean Temp. is the mean temperature; Max. Temp. is the maximum temperature; and RH is the relative humidity.
